# Combined use of AFP, CEA, CA125 and CAl9-9 improves the sensitivity for the diagnosis of gastric cancer

**DOI:** 10.1186/1471-230X-13-87

**Published:** 2013-05-14

**Authors:** Chao-Zhu He, Kun-He Zhang, Qing Li, Xiao-Hua Liu, Yan Hong, Nong-Hua Lv

**Affiliations:** 1 Department of Gastroenterology, The First Affiliated Hospital of Nanchang University; Jiangxi Institute of Gastroenterology & Hepatology, Nanchang, Jiangxi, 330006, China; 2 Nursing College of Nanchang University, Nanchang, Jiangxi, 330006, China

**Keywords:** Gastric cancer, Tumor markers, CEA, AFP, CA125, CA19-9

## Abstract

**Background:**

The detection of serum tumor marker becomes a common method for screening tumors. However, this method has not been widely used for routine gastric cancer screening. In this study we aimed to determine whether the combined use of tumor markers may increase the sensitivity for the diagnosis of gastric cancer.

**Methods:**

Serum AFP, CEA, CA125 and CA19-9 levels were measured in 149 patients with gastric cancer, 111 patients with benign gastric diseases and 124 healthy people, who visited the First Affiliated Hospital of Nanchang University from May 2011 to May 2012. Statistical analysis including receiver operating characteristic (ROC) curve, the area under the curve (AUC), and logistic regression analysis was performed to evaluate the diagnostic value of these markers on gastric cancer.

**Results:**

Serum levels of CEA, CA125, and CA19-9 in gastric cancer group were higher than that in the benign gastric disease group and the healthy control group (P <0.005). The sensitivity of AFP, CEA, CA125 and CA19-9 in the diagnosis of gastric cancer was 4.7-20.8% individually, and increased to 40.3% in combination. By using optimal cut-off value, the sensitivity of CEA, CA125, and CA19-9 for the diagnosis of gastric cancer was improved. Especially, the sensitivity of CEA increased to 58.4% and the sensitivity of combined use of four markers increased to 69.1%. The age and gender had no effects on the diagnostic value of these markers.

**Conclusions:**

The determination and application of optimal cut-off values based on ROC curve and logistic regression analysis could improve the diagnosis of gastric cancer based on common tumor markers.

## Background

It is difficult to differentiate the syndromes of gastric cancer and benign gastric diseases. Up to now, few effective biomarkers for gastric cancer have been applied in the clinic for the diagnosis of gastric cancer [1]. Currently, the diagnosis of gastric cancer mainly depends on invasive examination such as gastroscopy and biopsy. The detection of serum tumor marker is simple and easy and becomes a common clinical method for screening tumor. Tumor markers such as alpha fetoprotein (AFP), carcinoembryonic antigen (CEA), cancer antigen 125 (CA125) and cancer antigen 19–9 (CA19-9) have been widely used for the diagnosis of different types of cancers such as liver cancer, colorectal cancer and pancreatic cancer. However, when these markers are used individually for the diagnosis of gastric cancer, inconsistent results have been obtained [2-6].

A recent study reported that the use of a combination of Ki-67, Galectin-3, and PTTG can distinguish the benign and malignant thyroid tumor [7]. Therefore, we hypothesized that the combined use of tumor markers may avoid the inconsistence and increase the sensitivity for the diagnosis of gastric cancer. In this study we detected the serum levels of tumor markers AFP, CEA, CAl25 and CAl9-9 in 149 patients with gastric cancer, 111 patients with benign gastric diseases and 124 healthy people. Next we performed statistical analysis to compare the diagnostic value of these markers for gastric cancer. Our results showed that the determination of optimal cut-off values of these markers could increase the sensitivity for the diagnosis of gastric cancer.

## Methods

### Study subjects

Total 384 subjects were enrolled in this study who visited the First Affiliated Hospital of Nanchang University from May 2011 to May 2012, including patients with gastric cancer and benign gastric diseases, and healthy people who underwent physical examination. There were 149 patients in gastric cancer group (115 men, 34 women, age range 28 to 90 years old, average 59.1 years old). Among them, 30 patients had early gastric cancer and 119 patients had advanced gastric cancer. 71 patients had poorly differentiated adenocarcinoma, 72 patients had moderately differentiated adenocarcinoma, and 6 patients had highly differentiated adenocarcinoma. 70 patients had gastric antrum carcinoma, 61 patients had gastric body cancer, 13 patients had gastric cardia - bottom cancer, and 5 patients had multiple site cancer (more than two sites). There were 111 patients in benign gastric disease group (76 men, 35 women, age range 25 to 86 years old, average 52.9 years old). Among them, 24 patients had gastric ulcer, 53 patients had duodenal ulcer, 12 patients had complex ulcer, 17 patients had non-atrophic gastritis, and 5 patients had atrophic gastritis. 124 healthy people were included in the control group (72 men, 52 women, age range 37 to 83 years old, average 53.0 years old). Patients with gastric cancer and benign gastric diseases were diagnosed by endoscopy and confirmed by biopsy.

This study was performed in compliance with the Helsinki Declaration and according to a protocol approved by the Medical Ethics Committee of Nanchang University. All patients were informed about the study and gave their consent.

### Serum tumor marker detection

Serum AFP, CEA, CA125 and CA19-9 levels were detected by using Roche electrochemiluminescence instrument at Department of Nuclear Medicine of the First Affiliated Hospital of Nanchang University. The normal reference values were as follows: AFP≤7 ng/mL, CEA≤6.5 ng/mL, CAl25≤ 35 U/mL, CAl9-9≤27 U/mL.

### Statistical analysis

The data were expressed as mean ± standard deviation. The area under curve (AUC) of receiver operating characteristic (ROC) curve was used to evaluate the diagnostic value of serum tumor markers. Multivariate logistic regression analysis was used to establish the diagnostic mathematical model. On the basis of this model, the prediction value was calculated followed by ROC curve analysis. The statistical analysis was performed using SPSS17.0 statistical software (SPSS Inc., Chicago, IL, USA).

## Results

### The serum levels of CEA, CA125, CA19-9 and AFP in the study subjects

The demographic data such as age and gender and the test results of four serum tumor markers in gastric cancer group, benign gastric disease group and healthy control group were shown in Table 1. The average serum level and the positive rate of AFP, CEA, CA125 and CA19-9 in gastric cancer group were higher than that in the benign gastric disease group and the healthy control group.

**Table 1:** Serum levels of AFP CEA, CA125 and CA19-9 in the study subjects

	**Gastric cancer(n=149)**	**Benign gastric disease(n=111)**	**Healthy control(n=124)**	**Statistic test**
Age (Year)	59.1±11.5	52.9±17.4	53.0±9.6	F=10.129, P=0.000
Gender (Male/Female)	115/34	76/35	72/52	*χ*^2^=11.460, P=0.003
AFP (ng/mL)	3.8±16.8	3.3±18.6	2.4±1.3	F=0.332, P=0.718
CEA (ng/mL)	10.5±27.8	1.9±2.8	1.4±0.9	F=11.889, P=0.000
CA125 (U/mL)	20.1±32.7	11.6±17.5	9.9±7.2	F=7.895, P=0.002
CA19-9 (U/mL)	22.8±39.1	11.4±18.7	9.5±4.7	F=10.169, P=0.000
AFP Positive cases (%)	7 (4.7)	1 (0.9)	1 (0.8)	*χ*^2^=4.838, P=0.054
CEA Positive cases (%)	26 (17.4)	2 (1.8)	0 (0.0)	*χ*^2^=38.088, P=0.000
CA125 Positive cases (%)	21(14.1)	7 (6.3)	1 (0.8)	*χ*^2^=18.934, P=0.000
CA19-9 Positive cases (%)	31(20.8)	9 (8.1)	0 (0.0)	*χ*^2^=37.840, P=0.000
Combination Positive Cases (%)	60 (40.3)	14 (12.6)	2 (1.6)	*χ*^2^=74.443, P=0.000

### Positive rates of serum tumor markers in gastric cancer of different clinicopathological features

According to the stage, location and histological differentiation of gastric cancer, we subdivided gastric cancer group and calculated positive rates of 4 serum tumor markers with qualitative combined detection. The results showed that the positive rate of serum tumor markers in the advanced gastric cancer was higher than that in the early gastric cancer. The positive rate of AFP, CEA and CA19-9 with combined detection in the cardia cancer was higher than that in the other parts. However, there were no significant differences in the positive rate of serum tumor markers among gastric cancer with different differentiation (Table 2).

**Table 2:** Positive rates of 4 tumor markers with single and combined detection in gastric cancer with different stages, location and differentiation

	**Cases**	**Positive cases (%)**				
		**AFP**	**CEA**	**CA125**	**CA19-9**	**Qualitative combined**
Stage						
early stage	30	1 (3.3)	0 (0.0)	2 (6.7)	2 (6.7)	4 (13.3)
advanced stage	119	6 (5.0)	26 (21.8)	19 (16.0)	29 (24.4)	56 (47.1)
*χ*^2^		0.156	7.940	1.711	4.557	11.330
P		1.000	0.005	0.249	0.042	0.001
Location						
cardia	13	2 (15.4)	5 (38.5)	1 (7.7)	6 (46.2)	9 (69.2)
gastric body	61	0 (0.0)	12 (19.7)	10 (16.4)	7 (11.5)	21 (34.4)
gastric antrum	70	5 (7.1)	9 (12.9)	10 (14.3)	18 (25.7)	30 (42.9)
multi-site	5	0 (0.0)	0 (0.0)	0 (0.0)	0 (0.0)	0 (0.0)
*χ*^2^		7.504	6.275	1.529	10.630	8.965
P		0.056	0.098	0.696	0.016	0.026
Differentiation						
high	6	1 (16.7)	1 (16.7)	1 (16.7)	2 (33.3)	3 (50.0)
moderate	72	4 (5.6)	12 (16.7)	8 (11.1)	13 (18.1)	28 (38.9)
poor	71	2 (2.8)	13 (18.3)	12 (16.9)	16 (22.5)	29 (40.8)
*χ*^2^		2.599	0.070	1.024	1.031	0.303
P		0.268	0.930	0.735	0.605	0.839

### The association of serum tumor marker levels with the risk of gastric cancer

To evaluate the significance of serum tumor marker levels for the diagnosis of gastric cancer, we analyzed the association of serum tumor marker levels with gastric cancer in gastric cancer group. We got crude odds ratio (OR) after logistic regression analysis (Table 3). To exclude the possible effects of age and gender, we got adjusted odds ratio (ORa) after the adjustment of gender and age, and the results showed that CEA, CA125 and CA19-9 levels were positively correlated with the risk of gastric cancer (Table 3).

**Table 3:** The crude odds ratio (OR) and adjusted odds ratio (ORa) between serum tumor marker levels and the risk of gastric cancer

**Indicators**	**Crude odds ratio(OR)**	**P**	Adjusted odds ratio (ORa)*	**P**
AFP (ng/mL)	1.005 (0.991~1.019)	0.517	1.001 (0.987~1.015)	0.872
CEA (ng/mL)	1.460 (1.243~1.715)	0.000	1.389 (1.184~1.630)	0.000
CA125 (U/mL)	1.023 (1.010~1.036)	0.001	1.020 (1.006~1.034)	0.005
CA19-9 (U/mL)	1.029 (1.014~1.045)	0.000	1.026 (1.010~1.041)	0.001
combination	509.54 (90.92~2855.78)	0.000	341.87 (61.19~1910.10)	0.000

* Adjustment for age and gender.

### Use of normal cut-off values of serum tumor markers for the diagnosis of gastric cancer

We took the normal value of tumor marker serum level as cut-off value to determine the negative or positive of gastric cancer. The results showed that the use of single serum tumor marker had good specificity (96.2%-99.1%) but poor sensitivity (4.7%-20.8%) for the diagnosis of gastric cancer (Table 4). The sensitivity was improved with the combined use of serum tumor markers, but was still low (Table 4).

**Table 4:** Use of normal cut-off values of serum tumor markers for gastric cancer diagnosis

	**AFP**	**CEA**	**CA125**	**CA19-9**	**Combination**
Normal boundary value	≤7 ng/mL	≤6.5 ng/mL	≤35 U/mL	≤27 U/mL	-
AUC (95% CI)	0.519	0.583	0.553	0.585	0.667
	(0.459~	(0.523~	(0.493~	(0.525~	(0.609~0.726)
	0.579)	0.643)	0.614)	0.645)	
Sensitivity (%)	4.7	17.4	14.1	20.8	40.3
Specificity (%)	99.1	99.1	96.6	96.2	93.2
Accuracy (%)	62.5	67.4	64.6	66.9	72.7
Positive/Negative predictive value (%)	77.8/62.1	92.9/65.4	72.4/63.9	77.5/65.7	78.9/71.1
Positive/negative likelihood ratio	5.52/0.96	20.5/0.83	4.14/0.89	5.43/0.82	5.91/0.64

### Use of optimum cut-off values of serum tumor markers for the diagnosis of gastric cancer

Since the sensitivity in gastric cancer diagnosis with the use of normal cut-off values of four serum tumor markers was low, the potential in clinical application would be limited. Therefore, we analyzed the serum markers to obtain AUC of ROC curve, determined optimum diagnostic cut-off values, and then calculated their diagnostic values on gastric cancer. The results showed that the use of optimum boundary values could significantly improve the sensitivity in gastric cancer diagnosis with CEA and combination (58.4% and 47.7%, respectively) (Table 5).

**Table 5:** Use of optimal boundary values of serum tumor markers for gastric cancer diagnosis

	**AFP**	**CEA**	**CA125**	**CA19-9**	**Combination**
AUC (95% CI)	0.449	0.767	0.589	0.566	0.785
	(0.390~	(0.718~	(0.529~	(0.503~	(0.739~
	0.509)	0.816)	0.648)	0.630)	0.832)
Positive boundary value	≥7.29 ng/mL	≥2.24 ng/mL	≥22.90 U/mL	≥19.53 U/mL	≥0.419
Sensitivity (%)	4.7	58.4	19.5	30.2	47.7
Specificity (%)	99.1	83.4	95.7	92.8	91.1
Accuracy (%)	62.5	73.7	66.1	68.5	74.2
Positive/negative predictive value (%)	77.8/62.1	69.0/76.0	74.4/65.2	72.6/67.7	77.2/73.3
Positive/Negative likelihood ratio	5.52/0.96	3.52/0.50	4.57/0.84	4.18/0.75	5.33/0.58

## Discussion

In this study we analyzed the diagnostic value of four common clinical serum tumor markers AFP, CEA, CA125 and CA19-9 for gastric cancer. We compared the serum levels of these four markers in the gastric cancer group, benign gastric disease group and healthy control group, and found that the serum levels of all four markers were higher in gastric cancer group than in the benign gastric disease group and the healthy control group. Especially, the serum levels of CEA, CA125 and CA19-9 were significantly higher than in the benign gastric disease group and the healthy control group (P <0.005). In contrast, the serum levels of these four markers were not significantly different between the benign disease group and healthy control group (P = 0.581-0.844). These data suggest that these markers have diagnostic value for gastric cancer.

When we took the normal values of four markers as cut-off value to determine the negative or positive of the clinical specimens, we found that the specificity of four markers in the diagnosis of gastric cancer was more than 95%, when used individually, but the sensitivity was very low, ranging from 4.7% to 20.8%, and AUC was no more than 0.6. Thus single marker for clinical gastric cancer diagnosis is very limited [8]. When the four markers were combined, the sensitivity of the diagnosis of gastric cancer reached 40.3%, but was still not ideal.

To improve the sensitivity of gastric cancer diagnosis, we performed logistic regression analysis and used ROC curve to determine the optimum cut-off values. With the use of optimum cut-off values, for CEA the sensitivity was increased from 17.4% to 58.4% (the specificity was decreased from 99.1% to 83.4%), and AUC reached 0.767. These results are consistent with earlier studies performed in Chinese population [5,9].

Furthermore, we investigated the relationship between serum levels of tumor markers and clinicopathological features of gastric cancer. We found that the positive rates of CEA, CA19-9, and combined use of four markers were higher in the advanced gastric cancer than in the early gastric cancer. The positive rates of CA19-9 and combined use of four markers were higher in the cardia cancer than in the other parts of the gastric cancer. Our results are in agreement with previous reports that the stage and sites of gastric cancer would affect serum marker levels [10-12]. However, the positive rates of markers among the gastric cancer with different differentiation were not statistically significant, suggesting that the differentiation has little impact on gastric carcinoma marker level as reported previously [13].

In this study, the age and gender ratio of study subjects in the three groups were statistically significant. To avoid the possible bias caused by these factors, we analyzed the effects of age and gender on serum levels of the four markers in the diagnosis of gastric cancer. We performed multivariate logistic regression analysis and got ORa adjusted for age and gender. The results showed that the differences in age and gender could not change the diagnostic value of markers for gastric cancer.

## Conclusions

While the four common tumor markers when used individually has little clinical value in the diagnosis of gastric cancer, with the help of the ROC curve analysis and logistic regression analysis, we could determine the optimal cut-off values of the markers and improve the diagnosis of gastric cancer based on these markers. These results suggest that the optimal application of these common tumor markers could promote the clinical screening and diagnosis of gastric cancer.

## Competing interests

The authors declared that they have no competing interest.

## Authors’ contributions

KHZ, CZH and NHL designed the study; CZH, QL and XHL collected the samples; CZH and YH detected serum marker levels; KHZ and CZH performed statistical analysis; CZ H and KHZ drafted the manuscript. All authors read and approved the final manuscript.

## Pre-publication history

The pre-publication history for this paper can be accessed here:

http://www.biomedcentral.com/1471-230X/13/87/prepub

## References

[B1] Jackson C Cunningham D Oliveira J Gastric cancer: ESMO clinical recommendations for diagnosis, treatment and follow-up Ann Oncol 2009 20 Suppl 4 34 36 19454457 10.1093/annonc/mdp122

[B2] Haglund C Kuusela P Roberts P Jalanko H Tumour marker CA 125 in patients with digestive tract malignancies Scand J Clin Lab Invest 1991 51 3 265 270 10.3109/00365519109091613 1715601

[B3] Ychou M Duffour J Kramar A Clinical significance and prognostic Value of CA72-4 compared with CEA and CA19-9 in patients with gastric cancer Dis Markers 2000 16 3–4 105 110 11381189 10.1155/2000/595492 PMC3851416

[B4] Li Y Yang Y Lu M Predictive value of serum CEA, CA19-9 and CA72.4 in early diagnosis of recurrence after radical resection of gastric cancer Hepatogastroenterology 2011 58 112 2166 2170 22024091 10.5754/hge11753

[B5] Bornschein J Selgrad M Wex T Kuester D Malfertheiner P Serological assessment of gastric mucosal atrophy in gastric cancer BMC Gastroenterol 2012 12 10 10.1186/1471-230X-12-10 22289789 PMC3280182

[B6] Zur B Holdenrieder S Walgenbach-Brünagel G Albers E Stoffel-Wagner B Method comparison for determination of the tumor markers AFP, CEA, PSA and free PSA between Immulite 2000 XPI and Dimension Vista 1500 Clin Lab 2012 58 1–2 97 105 22372351

[B7] Cui W Lu X Zheng S Ma Y Liu X Zhang W The use of a combination of Ki-67, Galectin-3, and PTTG can distinguish the benign and malignant thyroid tumor Clin Lab 2012 58 5–6 419 426 22783570

[B8] Lai IR Lee WJ Huang MT Comparison of serum CA72-4, CEA, TPA, CA19-9 and CA125 levels in gastric cancer patients and correlation with recurrence Hepatogastroenterol 2002 49 1157 1160 12143226

[B9] Chen XZ Zhang WK Kun Y Correlation between serum CA724 and gastric cancer: multiple analyses based on Chinese population Mol Biol Rep 2012 39 9031 9039 10.1007/s11033-012-1774-x 22752725

[B10] Duraker N Celik AN The prognostic significance of preoperative serum CA 19–9 in patients with resectable gastric carcinoma: comparison with CEA J Surg Oncol 2001 76 4 266 271 10.1002/jso.1044 11320518

[B11] Fan B Xiong B Investigation of serum tumor markers in the diagnosis of gastric cancer Hepatogastroenterology 2011 58 105 239 245 21510322

[B12] Mihmanli M Dilege E Demir U The use of tumor markers as predictors of prognosis in gastric cancer Hepatogastroenterology 2004 51 59 1544 1547 15362797

[B13] Wobbes T Thomas CM Segers MF Evaluation of seven tumor markers (CA 50, CA 19–9, CA 19–9 TruQuant, CA 72–4, CA 195, carcinoembryonic antigen, and tissue polypeptide antigen) in the pretreatment sera of patients with gastric carcinoma Cancer 1992 69 8 2036 2041 10.1002/1097-0142(19920415)69:8<2036::AID-CNCR2820690805>3.0.CO;2-M 1544112

